# Cardiac Biomarkers are Associated with Incident Fracture Risk in Advanced Chronic Kidney Disease

**DOI:** 10.1007/s00223-024-01275-4

**Published:** 2024-08-20

**Authors:** Louise Aaltonen, Tapio Hellman, Roosa Lankinen, Markus Hakamäki, Kaj Metsärinne, Mikko Järvisalo

**Affiliations:** 1 Kidney Center, Department of Medicine, Turku University Hospital, PL 52, Kiinamyllynkatu 4-8, 20521 Turku, Finland; 2 Department of Medicine, Satakunta Central Hospital, Sairaalantie 3, 28500 Pori, Finland

**Keywords:** Chronic kidney disease, Fracture risk, Cardiovascular disease, Troponin T

## Abstract

Cardiovascular disease is associated with increased fracture risk in the general population. Few data exist on the association between cardiovascular health and incident fracture risk in patients with advanced CKD, a high-risk population for fractures. We aimed to assess the link between fracture risk and cardiovascular health in a prospective cohort of 210 patients with CKD stage G4–5. Incident fractures were recorded during a prospective follow-up of 5 years. Laboratory parameters, abdominal aortic calcification score, echocardiography, ultrasound assessment of brachial artery flow-mediated dilatation and carotid intima-media thickness, and maximal stress ergometry were obtained at baseline. A total of 51 fractures were observed in 40 (19%) patients during follow-up. In separate multivariable Cox proportional hazards models adjusted for age, gender, and baseline eGFR, TnT (HR 1.007, CI 95% 1.003–1.010, *p* < 0.001) and ProBNP (HR 1.000, CI 95% 1.000–1.000, *p* = 0.017) were associated with incident fractures and the association persisted after adjusting for coronary artery disease (CAD). The patients unable to perform the ergometry test had a higher risk of incident fractures compared to others (36.1% vs 15.5%, *p* = 0.009). A cardiovascular composite risk score summarizing TnT, ProBNP, and ergometry data was independently associated with incident fractures in a multivariable Cox model (HR 1.373, CI 95% 1.180–1.599, *p* < 0.001). Patients with the lowest score were observed with no fractures, while patients with the highest score were observed with a fracture risk of 40.5% during follow-up. Risk of incident fractures is associated with biomarkers of cardiovascular health and a composite cardiovascular risk score in patients with advanced CKD.

**Supplementary Information:**

The online version contains supplementary material available at 10.1007/s00223-024-01275-4.

## Introduction

Clinical manifestations of cardiovascular and cerebrovascular disease are associated with a markedly increased risk of incident bone fractures in the general population [1–3]. Furthermore, previous data indicate that fracture risk is associated with subclinical markers of atherosclerosis including endothelial dysfunction, carotid intima-media thickness (IMT) [4, 5], and abdominal aortic calcification score [6] in individuals without chronic kidney disease (CKD) even in the absence of clinical cardiovascular disease.

Fracture risk is high in patients with even mild forms of CKD and increases further with deteriorating kidney function reaching a four- to sixfold higher risk in end-stage kidney disease (ESKD) [7]. Impaired bone metabolism and associated fracture risk are multifactorial in CKD and remain under controversy and debate beyond that present in the general population. In the Stockholm CREAtinine Measurement project including 68764 individuals with confirmed stage G3–5 CKD, cardiovascular sequelae, namely, prior myocardial infarction, congestive heart failure, and cerebrovascular disease in addition to CKD stage, were associated with incident bone fractures, during a median follow-up of 2.7 years [8]. Impaired bone metabolism is linked to excess arterial calcification and aortic stiffening [9, 10], and vascular calcification has been shown to be associated with increased fracture risk in patients with ESKD [11]. Aside from vascular calcification, however, there are no previous data available, on the association between markers of cardiovascular health and subclinical cardiovascular disease and incident fracture risk in patients with advanced CKD. As the incidence of bone fractures is elevated in this highly comorbid patient population, it is important to examine factors associated with incident fracture risk to identify potential tools for individual risk stratification.

Therefore, we set out to examine the association between cardiovascular health and incident bone fractures using a wide range of laboratory and non-invasive cardiovascular risk assessment techniques in a prospective cohort of patients with CKD stage G4-5.

## Methods

The Chronic Arterial Disease, quality of life, and mortality in chronic KIDney injury (CADKID) study is a prospective follow-up study protocol assessing arterial disease, quality of life and mortality in patients with CKD stage G4–5. A total of 210 consecutive patients referred to the predialysis outpatient clinic of Turku University Hospital were recruited to the main study protocol between August 2013 and September 2017. The present study is a post hoc report from the CADKID study. A total of two hundred and ten patients were recruited from the predialysis outpatient clinic in the Kidney Center at Turku University Hospital. Inclusion criteria were estimated glomerular filtration rate (eGFR) < 30 ml/min per 1.73m^2^ using the Chronic Kidney Disease Epidemiology Collaboration (CKD-EPI) equation and age > 18 years.

Medications, relevant medical history, and documented fractures were gathered by the researchers from the electronic patient records of the research hospital. The fractures were divided into low and high energy fractures. Falling from height (> 1 m), road traffic accidents, and injuries due to external violence were considered high energy fractures, while other injuries such as falling from ground level were considered low energy fractures. Information was gathered during a follow-up time of 5 years from the time of recruitment. The study participants were followed up every 3–4 months, including laboratory parameters (analyzed by the laboratory (TYKSLAB) of the research hospital) and clinical status as per clinical guidelines [12]. All study patients resided in the catchment area of the research hospital and all incident fractures are referred to the research hospital for assessment and care. Dual-energy X-ray absorptiometry (DXA) was not part of the study protocol and performed as per the discretion of the attending clinician.

### Assessment of Cardiovascular Health

#### Abdominal Aortic Calcification (AAC) Score

Lateral lumbar radiography was performed using standard radiographic equipment. A validated score system by Kauppila et al. [13] was used to grade the calcification of the abdominal aorta. In this score system, the anterior and posterior aspects of the abdominal aorta in the region corresponding to the 1st to the 4th lumbar vertebra were analyzed in a total of eight segments. The degree of calcification per segment is scored between 0 and 3, so that the total score range is from 0 to 24. Individual aortic segments both from the anterior and the posterior walls were summed to get the anteroposterior severity score (0—24). All radiographs were analyzed independently by two researchers. The mean score was used in the analysis.

#### Maximal Stress Ergometry

All study participants underwent maximal bicycle stress ergometry, if they were in physical condition to perform the test. Altogether, 174 (82.9%) patients performed an incremental, symptom-limited cycling exercise test. The target speed was 60 rpm and it was reached after a warm-up phase of 30 s. The participants were encouraged to continue the test until exhaustion. An individual primary test workload was determined based on an estimated maximum workload (using as reference datasets of the Mini Suomi study [14]) and duration of the test workload increase/min (10, 15, or 20W) was accomplished automatically by the ergometer software. The percentage of the mean workload of the last 4 min of exercise (wlast4%) was obtained. Also heart rate and maximal oxygen uptake for all patients were obtained.

#### Echocardiography

The echocardiographic measures were obtained at baseline from a standardized transthoracic echocardiography performed by the Department of Clinical Physiology of Turku University Hospital before the stress ergometry. A commercially available ultrasound system (Vivid E9; GE Vingmed Ultrasound, Horten, Norway) with a 3.5-MHz phased-array transducer (M5S) was used. The systolic and diastolic function and dimensions of the left ventricle were measured, as well as left ventricular wall thicknesses, the left ventricular mass index, and left ventricular ejection fraction (EF).

#### Ultrasound Assessment of Brachial Artery Flow-Mediated Dilatation and IMT

Ultrasound methods employed and their reproducibility have been previously published in detail [15]. Briefly, for IMT measurements, the left common carotid artery ca. 1 cm proximal to the carotid bulb was scanned using B-mode and several images were saved digitally for later blinded offline analysis. To assess brachial flow-mediated dilatation (FMD), a measure of endothelial function, the right brachial artery diameter was measured both at rest and during the reactive hyperemia induced by inflation of a pneumatic tourniquet placed around the forearm, followed by release. The increase in vessel diameter after reactive hyperemia was expressed as the percentage relative to the resting scan.

As the focus of the present study was to assess the association between cardiovascular health and fracture risk, a composite risk score (3–12 points) was formed by summarizing TnT, ProBNP, and Wlast4% coded into four subgroups (1–4 points each) with a lower score indicating lower TnT or ProBNP or better ergometry test performance and a higher score indicating higher TnT or ProBNP or poorer ergometry test performance or the lack of ergometry test assessment due to poor physical condition.

### Statistical Analysis

The results for categorical covariates were reported as absolute and relative (percentage) frequencies and continuous covariates as mean ± standard deviation (SD) or median [inter-quartile range (IQR)] for normally distributed or non-parametric, respectively. Kolmogorov–Smirnov and Shapiro–Wilk tests were used to test normality in continuous covariates.

Categorical covariates were compared using the Fisher’s exact or Pearson × 2 test. The comparisons of continuous covariates were performed using Student’s *T* test for normally distributed covariates and Mann–Whitney *U* test for skewed covariates.

The associations between covariates of interest and incident fractures within 5 years of follow-up were explored separately with univariate Cox proportional hazards models. All covariates with an association at *p* < 0.05 level were entered in respective multivariable Cox proportional hazards models. The multivariable Cox models were performed by including age, gender and eGFR as covariates together with a single variable of interest (e.g., TnT or proBNP or urea etc.) in each respective model (Supplementary Table S1). With a moderate number of events (patients with fractures) in the cohort, adjustment for multiple covariates in the same multivariable model could not be done as including these variables in the models would have led to increased risk of multicollinearity.

To further explore the association between cardiovascular biomarkers and incident fracture risk, TnT and ProBNP were divided into quartiles (coded as one for the lowest quartile and four as the highest quartile of measurements) and Wlast4% measurements were divided into thirds. The patients who did not undergo the ergometry test formed the fourth Wlast4% subgroup. Next, the three subgroups (thirds) of patients with available Wlast4% measurements and the subgroup without ergometry test measurements were coded into four groups as follows: one as the highest third, two as the middle third, three as the lowest third of the available Wlast4% measurements, and four as the patients who did not undergo the ergometry test. Finally, the coded quartiles of TnT and ProBNP as well as the coded four subgroups of Wlast4% were summed together to form a composite risk score (3–12 points) covariate with the lowest value of three indicating patients with the Wlast4% performance in the highest third and TnT and ProBNP, both, in the lowest quartiles and the highest value of 12 indicating patients with no available Wlast4% measurement and TnT and ProBNP, both, in the highest quartiles.

The association between 5-year fracture risk and the composite risk score covariate was explored in a univariate Cox proportional hazards analysis and a multivariable Cox model adjusted with age, gender, and prior diagnosis of coronary artery disease (CAD) as covariates. The association between fracture risk and the composite risk score was further tested with a Kaplan–Meier plot and log rank test by dividing the composite risk score into four groups (one coded as the lowest score of three; two coded as the score 4–6; three coded as the score 7–9; and four coded as the score 10–12).

Finally, the association between incident fractures and all-cause mortality within 5 years of follow-up was assessed in a univariate Cox proportional hazards analysis.

All analyses were two-sided and *p* < 0.05 was considered significant. IBM SPSS statistics software version 27.0 was used for all analyses.

## Results

### Patient Characteristics

Demographic, clinical and laboratory characteristics, and medications of patients with and without an incident fracture within the 5-year follow-up are shown in Tables 1 and 2. During the 5-year follow-up, 67 patients died including 5 patients with a kidney transplant. At the time of death, 45 patients were on maintenance dialysis and 17 without the need for dialysis. A total of 176 patients started maintenance dialysis during follow-up and 77 received a transplant (including two pre-emptive transplantations). Altogether, 51 fractures were observed in 40 (19%) patients during follow-up. Eleven (28%) patients had two fractures. The majority of the patients with incident fractures [34 (85%)] experienced low energy fractures (Supplementary Table S2). Of the six patients with fractures categorized as high energy fractures, three experienced a fall from a height > 1 m, one experienced a violent attack, and two had a traffic accident. Five of the patients with high energy fractures were male and one female. Out of the 40 patients with incident fractures, 10 fractures occurred prior to initiation of renal replacement therapy, 24 in patients on dialysis and 6 after receiving a kidney transplant. By the end of follow-up, 67 (31.9%) patients had died. DXA was performed only in 21 (10%) patients and 9 (4.3%) were observed with osteoporosis and 8 (3.8%) with osteopenia during the study. Out of the 40 patients with incident fractures, 14 (35%) were assessed with DXA, 7 (17.5%) had osteoporosis, and 4 (10%) had osteopenia.

**Table 1:** Baseline characteristics of patients with and without incident fracture

	All patients	No fracture	Incident fracture	*p* value
Number of subjects	*n* = 210	*n* = 170	*n* = 40	
Demographics				
Age (years), mean (median)	62 (65)	61 (64)	67 (70)	0.02
Female subjects, *n* (%)	73 (34.8)	56 (32.9)	17 (42.5)	0.27
BMI, median (IQR)	27.7 (24.1–30.7)	27.7 (24.1–30.6)	27.6 (24.0–31.5)	0.87
History of smoking, *n* (%)	92 (43.8)	78 (46.7)	14 (35.0)	0.25
Hypertension, *n* (%)	205 (97.6)	167 (98,2)	38 (95.9)	0.24
Diabetes, *n* (%)	94 (44.8)	73 (42.9)	21 (52.5)	0.29
Peripheral artery disease, *n* (%)	37 (17.6)	29 (17.1)	8 (20.0)	0.65
Coronary artery disease, *n* (%)	35 (16.7)	31 (18.2)	4 (10.0)	0.25
History of heart failure, *n* (%)	48 (22.9)	36 (21.2)	12 (30.0)	0.30
History of arterial fibrillation, *n* (%)	41 (19.5)	28 (16.5)	13 (32.5)	0.03
Prior stroke, *n* (%)	23 (11.0)	18 (10.6)	5 (12.5)	0.78
Medication				
Aspirin, *n* (%)	59 (28.1)	46 (27.1)	12 (32.5)	0.56
Insulin, *n* (%)	71 (33.8)	55 (32.4)	16 (40.1)	0.36
Statin, *n* (%)	134 (63.8)	111 (65.3)	23 (57.5)	0.37
Calcium carbonate, *n* (%)	183 (87.1)	147 (86.5)	36 (90.0)	0.79
Calcium carbonate dose, mg	1000 (500–1000)	1000 (500–1000)	1000 (600–1000)	0.35
Phosphate binder, *n* (%)	26 (12.4)	21 (12.4)	5 (12.5)	1.0
Vitamin D, *n* (%)	172 (81.9)	138 (81.2)	34 (85.0)	0.66
Vitamin D, activated, *n* (%)	94 (44.8)	79 (46.5)	15 (37.5)	0.39
Cinacalcet, *n* (%)	6 (2.9)	4 (2.4)	2 (5.0)	0.32
Sodium bicarbonate, *n* (%)	124 (59.0)	105 (61.8)	19 (47.5)	0.11
Sodium bicarbonate dose, mg	2000 (1500–3000)	2000 (1500–3000)	2500 (1000–3500)	0.47

**Table 2:** Baseline biochemical, imaging, and ergometry data in patients with and without incident fracture

	No fracture	Incident fracture	*p* value
Number of subjects	*n* = 170	*n* = 40	
Biochemical data			
Creatinine (μmol/l)	398 (344–476)	384 (304–469)	0.13
eGFR ml/min	12 (10–15)	13 (12–15)	0.180
Urea (mmol/l)	22.2 (18.7–26.9)	23.5 (19.2–28.5)	0.19
Hemoglobin (g/l)	114 (106–123)	111 (104–119)	0.10
C-reactive protein (mg/l)	2 (1–4)	3 (1–9)	0.20
Erythrocyte sedimentation rate mm/h	30 (17–46)	36 (26–53)	0.10
Albumin (g/l)	34.9 (32.1–37.8)	34.0 (32.4–36.8)	0.45
Ionized calcium mmol/l)	1.21 (1.17–1.23)	1.21 (1.16–1.25)	0.95
Phosphorus (mmol/l)	1.44 (1.27–1.66)	1.47 (1.24–1.65)	0.94
Parathyroid hormone (ng/l)	181 (124–291)	170 (111–364)	0.86
Troponin T (ng/l)	33 (21–59)	852 (26–93)	0.02
proBNP (ng/l)	1040 (432–2850)	1720 (1015–5420)	< 0.01
pH	7.39 (7.35–7.41)	7.39 (7.37–7.43)	0.14
Bicarbonate (mmol/l)	22.3 (20.6–24.0)	22.5 (20.6–25.1)	0.35
Total cholesterol (mmol/l)	4.4 (3.5–5.0)	4.0 (3.5–4.8)	0.30
LDL cholesterol (mmol/l)	2.3 (1.7–2.9)	2.1 (1.4–2.9)	0.61
Triglycerides (mmol/l)	1.5 (1.2–2.2)	1.2 (1.0–1.9)	< 0.05
HDL cholesterol	1.10 (1.0–1.5)	1.25 (1.0–1.5)	0.89
Ferritin μg/l	196 (100–393)	344 (213–4835)	< 0.01
Transferrin saturation (%)	24.1 (19.1–29.5)	25.3 (18.2–30.6)	0.72
Thyroid-stimulating hormone (mU/I)	12 (10–15)	13 (12–15)	0.180
HbA1c (%)	105 (61.8)	19 (47.5)	0.11
Vascular imaging			
Abdominal aortic calcification score, AAC	6 (1–10)	8 (2–11)	0.32
Flow-mediated dilatation, FMD (mm) n = 172	*n* = 143	*n* = 29	
	4.1 (2.0–7.4)	3.2 (0.6–6.6)	0.197
Intima Media thickness of the carotid artery, CIMT (mm) n = 178	*n* = 147	*n* = 31	
	0.07 (0.06–0.09)	0.07 (0.06–0.08)	0.861
Stress ergometry and echocardiography, *n* = 174	*n* = 147	*n* = 27	
LV ejection fraction, (%)	65 (60–70)	66 (62–68)	0.64
LVEDD, (mm)	55 (50–58)	53 (50–56)	0.35
LV mass index	102 (86–123)	102 (89–139)	0.27
Wlast4 (W), (%)	57 (± 22)	49 (± 19)	0.08
Wlast4 (W)	82 (59–108)	77 (46–85)	0.03
Maximal heart rate, 1/min	120 (103–141)	109 (93–142)	0.18
MET, units	4.8 (3.8–5.8)	3.8 (3.2–5.1)	0.01
V0_2_max, ml/(kg*min)	16.7 (13.4–20.2)	13.3 (11.3–18.0)	0.01

Values are presented as median (IQR)

*Wlast4* mean workload of the last 4 min of maximal stress, *LVEDD* left ventricular end-diastolic diameter, *LF* left ventricle, *MET* metabolic equivalent of task

In the group-wise comparisons, patients with an incident fracture were older and had a higher prevalence of atrial fibrillation (AF) (Table 1) and performed more poorly in the maximal ergometry test (data available in 174 patients) (Table 2) and presented higher values of TnT and ProBNP and ferritin, but lower triglycerides. There were no significant differences between the groups concerning ongoing medications and assessed biomarkers for bone metabolism. Out of the cardiovascular biomarkers, TnT and proBNP were higher in the group with incident fractures, while AAC score, FMD, IMT, and echocardiographic measurements were similar between the groups (Table 2).

### Determinants of Fracture

Altogether older age, congestive heart failure, history of AF, lower hemoglobin or triglycerides, and higher Urea, TnT, ProBNP, and lower mean workload percentage of the last 4 min of maximal stress (Wlast4%) in the ergometry test were positively associated with incident fractures within the 5-year follow-up (Supplementary Table S1). Baseline AAC (HR 1.034, CI 95% 0.980–1.090, *p* = 0.219), FMD (HR 0.928, CI 95% 0.848–1.015, *p* = 0.101), or IMT (HR 0.003, CI 95% 0.000–113.403, *p* = 0.277)) were not associated with fractures during follow-up. In the multivariable proportional hazards models adjusted for age, sex, and eGFR as covariates in addition to a single variable of interest in each respective model, TnT (HR 1.007, CI 95% 1.003–1.010, *p* < 0.001), hemoglobin (HR 0.960, CI 95% 0.932–0.989, *p* = 0.007), Urea (HR 1.057, CI 95% 1.012–1.103, *p* = 0.013), and ProBNP (HR 1.000, CI 95% 1.000–1.000, *p* = 0.017) remained significantly associated with fracture risk at 5 years of follow-up. In a further analysis, TnT (*p* < 0.001) and ProBNP (*p* = 0.015) remained independently associated with fracture risk in a multivariable Cox model adjusted with the prior diagnosis of CAD.

In the 174 patients who performed the stress ergometry test, Wlast4 (HR 0.988, CI 95% 0.975–1.002, *p* = 0.085) or Wlast4% (HR 0.982, CI 95% 0.964–1.001, *p* = 0.057) were not associated with the risk of incident fractures in the adjusted multivariable Cox proportional hazards model. Out of the 36 patients who did not undergo maximal stress ergometry, 13 (36.1%) were observed with an incident fracture during the study and these patients had a higher incidence of fractures compared to those who attended the ergometry test (36.1% vs 15.5%, *p* = 0.009). Similarly, mortality was higher in patients in whom the ergometry test was not performed compared to those who underwent the ergometry test (61.1% vs 25.9%, *p* < 0.001).

**Fig. 1:**
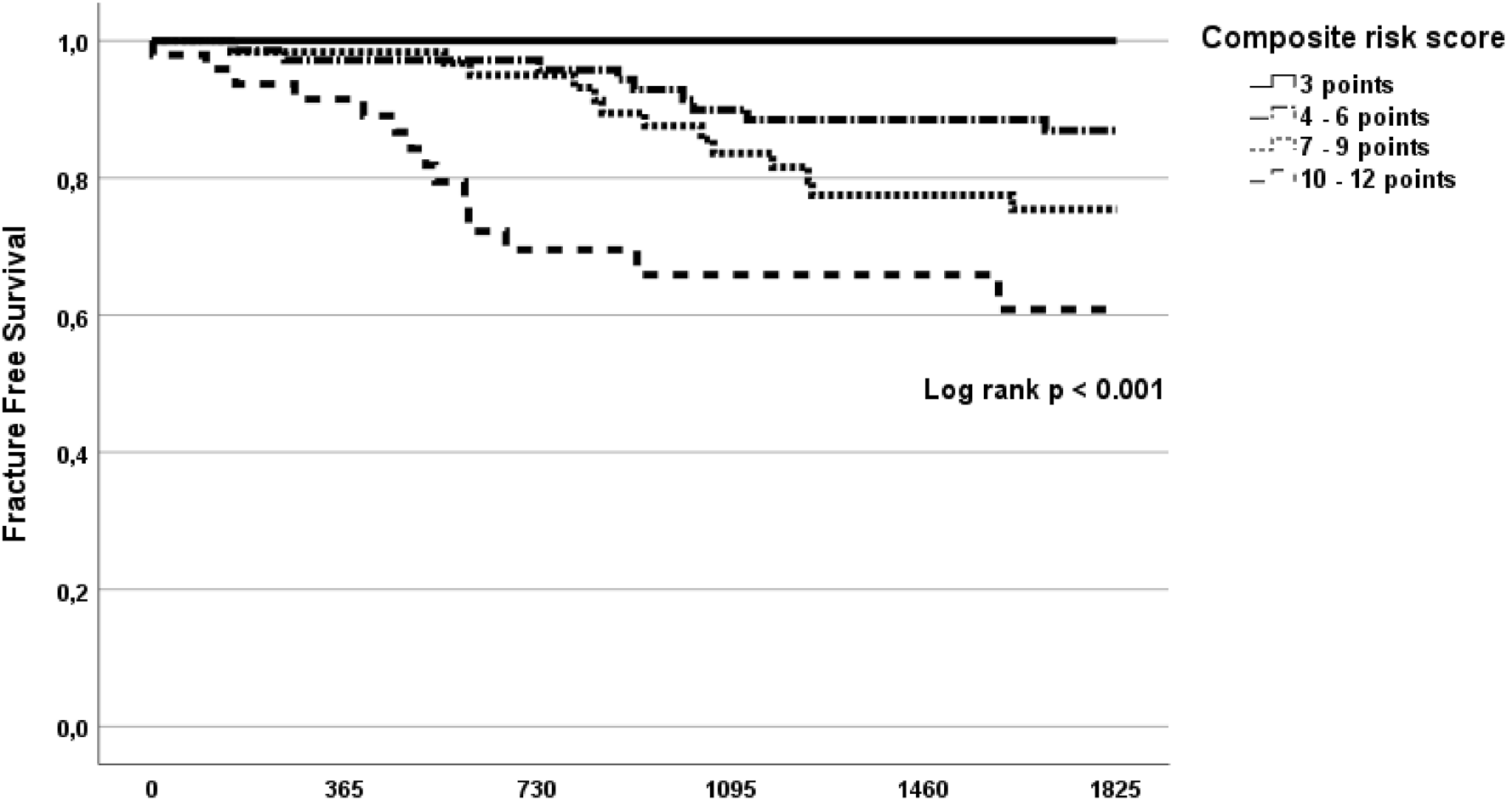
Kaplan–Meier plot with a log-rank test depicting the association between the composite risk score in quartiles and incident fractures

The patients with the lowest score were observed with no fractures while the patients with the highest score (10–12 points) were observed with fracture risk of 40.5% (Table 3). The relationship between the composite risk score and fracture risk within 5-year follow-up is depicted in Fig. 1. The composite cardiovascular risk score was significantly associated with incident fractures within 5-year follow-up in a univariate Cox proportional hazards analysis (HR 1.353, CI 95% 1.175–1558, *p* < 0.001) as well as in a multivariable Cox model (HR 1.373, CI 95% 1.180–1.599, *p* < 0.001) including age, gender, and previous diagnosis of clinical CAD as covariates. The multivariable association between the composite risk score and incident fractures remained similar, when patients (*n* = 6) with high energy fractures were excluded from the analysis (HR 1.366, CI 95% 1.164–1.603, *p* < 0.001).

**Table 3:** Distribution of composite risk score in patients with and without incident fracture

	No fracture *n* = 160	Incident fracture *n* = 37
Composite risk score: 3 points	15 (9.4)	0 (0)
Composite risk score: 4–6 points	62 (38.8)	9 (24.3)
Composite risk score: 7–9 points	49 (30.6)	13 (35.1)
Composite risk score: 10–12 points	34 (21.3)	15 (40.5)

Data missing in 13/210 (6.2%) patient due to unavailable TnT or ProBNP measurements

The composite risk score was calculated by summarizing TnT and ProBNP quartiles (coded as 1–4 points from the lowest to the highest quartile of measurements) and four subgroups of Wlast4% (coded as one point for the highest third, two points for the middle third, three points for the lowest third of the available Wlast4% measurements, and four points for the patients who did not undergo the ergometry test). The score ranges between 3 and 12 points with three points indicating measurements in the lowest TnT and ProBNP quartiles and ergometry test performance in the highest third of Wlast4% measurements and 12 points indicating TnT and ProBNP measurements in the highest quartile and the lack of ergometry test assessment

Incident fractures were not associated with mortality in univariate Cox proportional hazards analysis (*p* = 0.426).

## Discussion

The present study shows that a composite cardiovascular risk score is associated with increased incident fractures in patients with CKD stage G4–5. Patients with a low cardiovascular risk score presented with no fractures, whereas, 40.5% of patients with high cardiovascular risk experienced incident fractures during the 5-year follow-up. The association between the cardiovascular risk score and fractures remained significant after adjusting for prior CAD diagnosis in addition to age and gender. Moreover, incident fractures were independently associated with elevated troponin T and ProBNP. This is to our knowledge the first study to report such associations between markers of cardiovascular risk and incident fractures in patients with advanced CKD.

An increasing amount of data suggest an association between cardiovascular disease, atherosclerosis, and fracture risk in the general population [1, 2, 16]. Recently, Renjithal et al. demonstrated a link between attenuated BMD assessed with DXA and cardiovascular events [17]. Osteoporosis and cardiovascular disease share many common risk factors, such as diabetes, age, vitamin D deficiency, smoking, postmenopausal state, and low-grade inflammation [2] and these risk factors are frequently present also in patients with CKD. There are, however, emerging data of a common pathophysiological mechanism behind the disorders [18]. Several clinical studies have confirmed an association between vascular calcification, an established independent risk factor for cardiovascular disease associated with CKD, and low bone mass [19, 20]. Furthermore, Naves et al. showed in a prospective study that progression of vascular calcification is associated with fracture risk and decline in BMD in men and women with normal kidney function [6]. In advanced CKD, the disturbance in the bone-vascular axis is even more complex and still a matter of controversy. Both bone mineralization and pathologic development of vascular calcification are actively regulated and there seems to be a co-existence of bone loss and progressive vascular calcification in CKD patients [21]. Even though several studies have addressed this issue, there are very little and partly inconsistent data on fracture risk and clinical cardiovascular disease in patients with advanced CKD.

Cardiac biomarkers TnT and proBNP have been shown to be associated with future major adverse cardiovascular events and death in CKD [22–24], including the CADKID study cohort [25]. However, there are no previous data available showing that these markers are associated with incident fracture risk. In the present study, TnT and proBNP were associated with incident fractures after adjusting for age, gender, and baseline eGFR as well as prior diagnosis of CAD. As cardiac biomarkers are associated with all-cause and cardiovascular mortality in CKD, they may reflect the overall morbidity of these patients even in the absence of clinical cardiovascular disease and therefore make them prone to incident fractures [26, 27]. In patients with ESKD, the ongoing disturbance in mineral and bone metabolism leads to increased levels of fibroblast growth factor 23 (FGF-23) which has also been associated with left ventricular hypertrophy and increased troponin T levels in hemodialysis patients, suggesting a link between bone health and TnT levels [28].

In the present study, no association was observed between fracture risk and markers of subclinical atherosclerosis, such as IMT and FMD or AAC score. Previous studies in the general population have shown an association between IMT and fracture risk [4, 5], whereas data concerning FMD and fracture risk are inconsistent [27, 28]. In our cohort of patients with advanced CKD, patients both with and without fractures had rather high AAC scores, indicating severe vascular calcification. According to our current findings, TnT, ProBNP, and the composite cardiovascular risk score, potentially more robust indicators of poor vascular health and accumulated vascular damage, are more indicative of incident fracture risk compared to imaging of subclinical cardiovascular disease or echocardiographic indices. However, there were no significant differences in the prevalence of CAD, heart failure, or peripheral artery disease between patients with or without incident fractures. This finding is in contrast with prior evidence and may partly be related to limited sample size [29].

Fracture risk is high in ESKD and linked to increased morbidity and mortality [30]. Increased mortality has also been observed in ESKD patients with low BMD [31]. In this study population, fractures were not associated with increased mortality. Poor physical performance is common in ESKD patients and has been associated with low BMD, falls, and fractures in patients with or without kidney dysfunction [32, 33]. Importantly, the fracture risk was twice as high in patients who did not undergo the ergometry test compared to those who did. This indicates that selection bias might explain the difference as the frailest patients with the poorest prognosis were not able to perform the test. Concordantly, mortality was substantially higher in the patients in whom the ergometry test was not performed. Inability to perform a preoperative bicycle ergometry stress test has been associated with cardiovascular disease and mortality in lung cancer patients subjected to major operative care [34]. To our knowledge, the outcomes of ESKD patients unable to undergo ergometry stress testing have not been studied previously. We, however, believe that the failure to perform an ergometry test effectively reflects high risk for adverse bone and cardiovascular outcomes as well as poor survival. For this reason, the inclusion of the inability to perform and the exercise capacity assessed with the ergometry test into the composite cardiovascular risk score is intuitive and indirectly serves as a robust measure of frailty in these patients. Our study shows that incident long-term fracture risk in ESKD patients could be effectively stratified using few simple readily available biomarkers and the composite risk score might be applicable in assessing the risk of fractures, whether the patients are physically fit enough to perform the ergometry test or not.

The current study has several limitations related to its observational, single-center nature. The sample size was relatively small and there was a selection bias in attending stress ergometry, as not all patients could perform the test. However, the inability to undergo the ergometry test was associated with high risk of adverse bone outcomes and poor survival justifying the inclusion of these patients into the composite risk score. DXA was performed in only 10% of the study population. However, the study population was extensively assessed using several cardiovascular imaging and biochemical methods to examine the potential association between subclinical cardiovascular disease and incident fracture risk in this highly comorbid group of patients.

## Conclusion

Cardiac biomarkers and a composite cardiovascular risk score including biomarkers and maximal ergometry stress testing are associated with incident bone fractures in patients with CKD stage G4–5. Further studies are warranted to evaluate whether the risk assessment for individual fracture risk should include assessment of cardiac biomarkers.

## Supplementary Information

Below is the link to the electronic supplementary material.

Supplementary file1 (DOCX 17 KB)

Supplementary file2 (DOCX 15 KB)
