# Supplementary material for: Cardiac Biomarkers are Associated with Incident Fracture Risk in Advanced Chronic Kidney Disease
Source: Calcif Tissue Int. 2024 Aug 20;115(5):516–24. doi: 10.1007/s00223-024-01275-4 (PMC11531441; doi:10.1007/s00223-024-01275-4)
Supplement: Supplementary file 1 — Supplementary file1 (DOCX 17 KB) [file 223_2024_1275_MOESM1_ESM.docx]

| **Supplementary Table S1.** | **Univarite models** | | | **Multivariable models** | | |
| --- | --- | --- | --- | --- | --- | --- |
| **Covariates** | HR | CI95% | **P-value** | HR | CI95% | **P-value** |
| Age | 1.038 | 1.012-1.065 | 0.005 | NA | NA | NA |
| Heart failure | 2.059 | 1.044-4.059 | 0.037 | 1.477 | 0.723-3.014 | 0.284 |
| Prior diagnosis of AF | 2.579 | 1.326-5.017 | 0.005 | 1.604 | 0.750-3.433 | 0.223 |
| Hemoglobin (g/l) | 0.970 | 0.945-0.996 | 0.024 | 0.960 | 0.932-0.989 | 0.007 |
| Urea (mmol/l) | 1.053 | 1.005-1.103 | 0.030 | 1.057 | 1.012-1.103 | 0.013 |
| Troponin T (ng/l) | 1.007 | 1.004-1.011 | <0.001 | 1.007 | 1.003-1.010 | <0.001 |
| ProBNP (ng/l) | 1.000 | 1.000-1.000 | 0.012 | 1.000 | 1.000-1.000 | 0.017 |
| Triglycerides (mmol/l) | 0.613 | 0.376-0.997 | 0.049 | 0.631 | 0.380-1.048 | 0.075 |
| Wlast4 (W) | 0.984 | 0.973-0.996 | 0.009 | 0.988 | 0.975-1.002 | 0.085 |
| Wlast4 (W) % | 0.979 | 0.962-0.998 | 0.026 | 0.982 | 0.964-1.001 | 0.057 |
| MET, units | 0.635 | 0.462-0.873 | 0.005 | 0.675 | 0.477-0.956 | 0.027 |
| V0_2_max, ml/(kg*min) | 0.878 | 0.802-0.962 | 0.005 | 0.894 | 0.809-0.988 | 0.028 |

Supplementary Table S1. Univariate and multivariable associations between covariates of interest and incident fractures. Each respective multivariable Cox proportional hazard model included a single covariate of interest listed in the table together with age, gender and eGFR entered as covariates for all models.
