# Supplementary material for: Cardiac Biomarkers are Associated with Incident Fracture Risk in Advanced Chronic Kidney Disease
Source: Calcif Tissue Int. 2024 Aug 20;115(5):516–24. doi: 10.1007/s00223-024-01275-4 (PMC11531441; doi:10.1007/s00223-024-01275-4)
Supplement: Supplementary file 2 — Supplementary file2 (DOCX 15 KB) [file 223_2024_1275_MOESM2_ESM.docx]

| **Fractures** | **Total** | **Predialysis** | | **Dialysis** | | **Post transplantation** | |
| --- | --- | --- | --- | --- | --- | --- | --- |
|  | **n=51** | **Low energy** | **High energy** | **Low energy** | **High energy** | **Low energy** | **High energy** |
| **Vertebrae** | 7 | 2 |  | 2 | 2 | 1 |  |
| **Hip** | 10 | 4 |  | 6 |  |  |  |
| **Pelvis** | 6 | 2 |  | 3 | 1 |  |  |
| **Humerus** | 5 | 1 | 1 | 3 |  |  |  |
| **Costae** | 3 |  | 1 | 2 |  |  |  |
| **Femur** | 1 | 1 |  |  |  |  |  |
| **Ulna,Radius** | 4 | 1 |  | 3 |  |  |  |
| **Tibia, Fibula** | 8 | 1 |  | 4 |  | 2 | 1 |
| **Metatarsals** | 3 |  | 1 | 1 |  | 1 |  |
| **Metacarpals** | 2 | 1 |  | 1 |  |  |  |
| **Proximal phalanges** | 2 |  |  | 1 |  |  | 1 |

Table S2. Incident fractures during the 5-year follow-up by chronic kidney disease treatment modality.
